# Omentin-1 rs2274907 and resistin rs1862513 polymorphisms influence genetic susceptibility to nonalcoholic fatty liver disease

**Published:** 2016-03

**Authors:** Leila Kohan, Mehrnush Safarpur, Hamed Abdollahi

**Affiliations:** 1 Department of Biology, Arsanjan branch, Islamic Azad University, Arsanjan, Iran; 2 Yong Researchers and Elite Club, Islamic Azad University, Arsanjan Branch, Arsanjan, Iran

**Keywords:** Non-alcoholic fatty liver disease, Polymorphism, ITLN, Resistin

## Abstract

Nonalcoholic fatty liver disease (NAFLD) is an obesity-associated disease and dysregulation of adipokines has an important role in its development. Omentin-1 (ITLN1 protein) and resistin are two adipokine secreted from adipose tissue. Single nucleotide polymorphisms in the adipokine genes may affect expression and activity of the adipokine, and thus play a contributory role in NAFLD pathogenesis. The aim of the present study was to investigate the association between omentin-1 rs2274907 (326A/T) and resistin rs1862513 (-420 C/G) polymorphisms and risk of NAFLD in Iranian patients. This case-control study was done on 282 subjects included 94 patients with NAFLD and 188 healthy peoples. The genotypes were determined using PCR-RFLP method. The frequency of omentin-1 AT genotype in patients with NAFLD was significantly different from that in the control (OR=2.3, 95% CI: 1.3-3.8, P=0.003). A significant association was observed between NAFLD and the GG genotype regarding resistin rs1862513 polymorphism (OR=2.3, 95% CI: 1.1-4.8, P=0.03). In conclusion, Omentin-1 rs2274907 and resistin rs1862513 polymorphisms might be a candidate genetic factor for susceptibility to NAFLD.

## INTRODUCTION

Nonalcoholic fatty liver disease (NAFLD) is one of the most prevalent types of liver diseases in western countries [1]. It can progress to advanced liver fibrosis, cirrhosis and hepatocellular carcinoma [2]. The rate of NAFLD is strongly linked to obesity, insulin resistance and other components of the metabolic syndrome [3]. In fact, although the development of NAFLD is strongly linked to obesity and insulin resistance, there are obese individuals who do not have NAFLD, and since NAFLD can occur in normal-weight individuals with a normal metabolic profile, thus multiple genetic and environmental factors should be involved in its development [4]. Adipokines are the fat-derived hormones released from adipose tissue. Levels of adipokines in NAFLD patients were found to be different compared to controls [5, 6]. Omentin-1 and resistin are recently described as secretory adipokine of adipose tissue [7]. Omentin-1 (intelectin-1: *ITLN1*, OMIM: 609873) has been identified as a major visceral (omental) fat secretory adipokine. This adipokine may act as an endocrine factor affecting muscles, liver and omental adipose depot; it enhances insulin sensitivity and glucose metabolism [8]. Recently, it was found that serum omentin-1 is elevated in patients with fatty liver diseases and it represents an independent predictor for hepatocyte ballooning in these patients [6]. Resistin (*RETN*, OMIM: 605565), an adipokine belongs to the recently described resistin-like molecule family of small cysteine-rich secreted proteins [9]. It has also been suggested as a link between obesity and insulin resistance [10]. Insulin resistance, through inhibition of lipid oxidation and increased fatty acid and triglyceride synthesis, is believed to be a key factor in the development of fatty liver. [11]. In NAFLD, concentrations of resistin were higher than in controls and positively correlated with liver inflammation and fibrosis severity, but this was not consistent in all undertaken studies [12, 13]. 

The aim of the present study was to evaluate the impact of *ITLN1*rs2274907 (326A/T) and *RETN* rs1862513 (-420C/G) gene polymorphisms on the risk of NAFLD in a sample of the Iranian population.

## MATERIALS AND METHODS

**Participants: **A total of 282 blood samples was collected from June 2013 to February 2014. Informed consent was obtained from all blood donor participants and the study was performed in Shiraz city, located in southwest Iran. The study groups consisted of 188 healthy participants (98 men and 90 women, mean age ± SD: 45.9 ±14) matched with the 94 NAFLD patients (49 men and 45 women, mean age ± SD: 43 ±11.9) according to age (±5) and gender. Body mass index (BMI) was calculated as weight (in kg) divided by (height)^2^ (in m^2^).

NAFLD diagnosis was based on clinical symptoms, sonogeraphic and laboratory findings. Patients with viral hepatitis B and C, autoimmune liver diseases, hemochromatosis, Wilson disease, alcohol intake of more than 100g/week, and chronic drug consumption were excluded from the study. The study was approved by the local ethical committee of Shiraz University of medical sciences.

**DNA extraction and genotyping:** Genomic DNA was prepared from whole venous blood using a commercially available DNA isolation kit (Arash Teb, Iran).The genotyping was performed by PCR-RFLP method. The PCR reaction mixture contained 50-100 ng DNA, 0.5 μL dNTPs 10 mM, 0.75 μL MgCl_2_ 50 mM, 1μL of each primers (10pm/μL) and 0.3 U Taq DNA polymerase 5 U/μL (Cinagen, Iran) in a 25μL mixture. The sequence of the primers was listed in Table 1.

PCR was performed under the following conditions: 4 min at 95°C followed by 40 cycles of 1 min at 94°C, 1 min at 62°C for rs2274907, 1 min at 55°C for rs1862513, and 1 min at 72°C, with a final step 5 min at 72°C. For Omentin-1 rs2274907, The PCR products were then digested overnight with 10 U of *Xmil* (*AccI*) restriction endonuclease (Fermentas, Germany), which cut the amplified DNA into 274 bp and 197 bp fragments in the presence of the T allele, while the A variant remained uncut showing the 471 bp PCR product. For rs1862513 polymorphism, *BpiI *restriction analysis showed 327 bp and 207 bp fragments for C allele and one fragment of 533 bp for G allele.

**Table 1:** The primers used for PCR method

**Polymorphisms**	**Sequences **	**References**
***ITLN1*** **rs2274907**	F: 5´-GAGCCTTTAGGCCATGTCTCT-3´ R: 5´-CTCTCTTCTTCTCCAGCCCAT-3´	14
***RETN*** ** rs1862513**	F: 5´-TGTCATTCTCACCCAGAGACA-3´ R: 5´-TGGGCTCAGCTAACCAAATC-3´	15

**Statistical analysis**: Statistical analysis was performed using SPSS 19.0 software. Hardy-Weinberg analysis was performed to compare the observed and expected genotype frequencies using χ^2^ test. The association of *ITLN1*rs2274907 and *RETN* rs1862513 genotypes and risk of NAFLD was estimated by odds ratio (OR) and 95% confidence intervals (CIs) calculated (*P*<0.05 was considered statistically significant). Unconditional logistic regression was used to calculate OR and 95% CI for the various genotypes after adjusting for BMI, because this characteristic was significant at *P*<0.05.

## RESULTS AND DISCUSSION

The anthropometric parameters of the patient and control groups are summarized in Table 2. There was a significant difference in Weight (*P*<0.001) and BMI (*P*=0.01) between NAFLD patients and control group. 

**Table 2:** Anthropometric characteristics in NAFLD patients and controls groups

	**NAFLD**	**Controls**	***P***
**Height (cm)**	10.6±167.9	165.3±10.7	0.059
**Weight (kg)**	80.8±16.2	72.4±13.6	<0.001
**BMI (kg/m** ^2^ **)**	28.5±5.4	27±4.5	0.010

The genotype and allele frequencies of *ITLN1*rs2274907 polymorphism are shown in Table 3. The *ITLN1*rs2274907 polymorphism in controls was in Hardy-Weinberg equilibrium (χ^2^=1.58, *df*=1, *P*=0.208). Significant differences were observed in AT genotype frequency between case and control groups (OR=2.3, 95%CI=1.3-3.8, *P*=0.003). TT genotype frequency was 5 (5.3%) and 7 (3.7%) in case and control groups, respectively. There was no significant association between TT genotype and the risk of NAFLD (OR=2.1, 95% CI=0.63-7.18, *P*=0.22). In dominant effect of the T allele (AT+TT *vs* AA), AT+TT genotypes were associated with NAFLD risk (OR=2.2, 95% CI=1.3-3.8, *P*=0.002). 

**Table 3:** The genotypes of *ITLN1*rs2274907 polymorphism in case and control groups

**Genotypes**	**NAFLD N (%)**	**Control N (%)**	**OR** * ** (95%CI)**	***P*** *
**AA**	37 (39.4)	108 (57.5)	1 (Reference)	-
**AT**	52 (55.3)	73 (38.8)	2.3 (1.33-3.84)	0.003
**TT**	5 (5.3)	7 (3.7)	2.1 (0.63-7.18)	0.220
**AT+TT**	57 (60.6)	80 (42.5)	2.24 (1.33-3.77)	0.002

* adjusted for BMI

As shown in Table 4, a significant difference was observed in GG genotype frequencies between NAFLD and control groups regarding *RETN* rs1862513 polymorphism (OR=2.3, 95% CI=1.1-4.8, *P*=0.03). It should be noted that the genotypes of rs1862513 polymorphism in controls were in Hardy-Weinberg equilibrium (χ^2^=2.45, *df*=2, *P*=0.117).

**Table 4:** The genotypes of *RETN* rs1862513 polymorphism in case and control groups

**Genotypes**	**NAFLD N (%)**	**Control N (%)**	**OR** * ** (95%CI)**	***P*** *
**CC**	25 (26.6)	64 (34)	1 (Reference)	-
**CG**	47 (50)	100 (53.2)	1.2 (0.65-2.14)	0.596
**GG**	22 (23.4)	24 (12.8)	2.3 (1.11-4.82)	0.030

* adjusted for BMI

NAFLD is a complex metabolic condition in which both lifestyle and genetic factors have a pathogenic role. In complex diseases, several or many different genes interact with environmental factors in determining disease presence or its phenotype [16]. Initial evidence for a genetic component to NAFLD comes from familial clustering studies [17] and the ethnic variation in NAFLD prevalence [18]. Genes that are candidates for study in NAFLD have included genes influencing insulin resistance, fatty acid metabolism, oxidative stress, immune regulation and fibrosis development [15]. Various genetic single-nucleotide polymorphisms have been investigated in NAFLD including single-nucleotide polymorphisms in the adiponectin [19, 20], *IL-6* [21], *TNFα* [22] and *ApoE* [23] genes. The present study showed that there was a significant difference between NAFLD patients and control subjects regarding *ITLN1* rs2274907 and *RETN* rs1862513 gene polymorphisms. Recent studies show that omentin-1 is elevated in patients with liver cirrhosis [24] and omentin-1 level is significantly higher in patients with nonalcoholic fatty liver disease than in healthy controls [6]. In several studies, the relationship between rs2274907 polymorphism in omentin-1 gene and diseases such as diabetes [13], rheumatoid arthritis [25], psoriasis [26], and coronary artery disease [27] was investigated. 

Adult NAFLD patients display increased serum resistin values [12]. Pagano et al. (2006) showed that serum resistin was significantly higher in patients with NAFLD compared to healthy controls [11]. Zhang et al. (2013) investigated the relationship between the resistin intronic +299G/A polymorphism and nonalcoholic fatty liver disease (NAFLD) in patients with type 2 diabetes mellitus (T2DM) and reported that the resistin +299AA genotype may be associated with increase in the risk of the NAFLD development in T2DM patients [28]. Recently, Zhang et al (2015) reported that Resistin -420 G/C, glutathione peroxidase-1 Pro198Leu and cigarette smoking are three risk factors for NAFLD and have a significant additive effect on NAFLD risk [29]. 

Our results showed significant association between omentin-1 rs2274907 and *RETN* rs1862513 gene polymorphisms and NAFLD risk in Iranian population. One limitation of this study is its relatively small sample size. Therefore, larger studies with different ethnicities are required to confirm our finding.

## Notes

**Acknowledgments:** We acknowledge Dr. Mohamad Rahemi (Professor of Gastroenterology and Hepatology) for aid to provide NAFLD samples. Also, the authors would like to thank all participants who willingly contributed to the study.
